# Passive Transfer of Sera from ALS Patients with Identified Mutations Evokes an Increased Synaptic Vesicle Number and Elevation of Calcium Levels in Motor Axon Terminals, Similar to Sera from Sporadic Patients

**DOI:** 10.3390/ijms21155566

**Published:** 2020-08-03

**Authors:** Valéria Meszlényi, Roland Patai, Tamás F. Polgár, Bernát Nógrádi, Laura Körmöczy, Rebeka Kristóf, Krisztina Spisák, Kornélia Tripolszki, Márta Széll, Izabella Obál, József I. Engelhardt, László Siklós

**Affiliations:** 1 Biological Research Centre, Institute of Biophysics, 62 Temesvári krt., H-6726 Szeged, Hungary; mesval13@gmail.com (V.M.); patai.roland@brc.hu (R.P.); polgar.tamas@brc.hu (T.F.P.); bernatnogradi@gmail.com (B.N.); laura.kormoci96@gmail.com (L.K.); crebby32@gmail.com (R.K.); spisakkrisztina96@gmail.com (K.S.); 2 Foundation for the Future of Biomedical Sciences in Szeged, Szeged Scientists Academy, 15 Lechner tér, H-6721 Szeged, Hungary; 3 Department of Medical Genetics, University of Szeged, 4/B Szőkefalvi-Nagy Béla u., H-6720 Szeged, Hungary; tripolszki.kornelia@med.u-szeged.hu (K.T.); szell.marta@med.u-szeged.hu (M.S.); 4 Dermatological Research Group, University of Szeged, Hungarian Academy of Sciences, 4/B Szőkefalvi-Nagy Béla u., H-6720 Szeged, Hungary; 5 Department of Neurology, Aalborg University Hospital, 15 Skovvej Sdr., DK-9000 Aalborg, Denmark; obalizabella@yahoo.com; 6 Department of Neurology, University of Szeged, 6 Semmelweis u., H-6725 Szeged, Hungary; eji48dec9@yahoo.com

**Keywords:** ALS, passive transfer, intracellular calcium, synaptic vesicles, SOD1 mutation, C9ORF72 mutation

## Abstract

Previously, we demonstrated increased calcium levels and synaptic vesicle densities in the motor axon terminals (MATs) of sporadic amyotrophic lateral sclerosis (ALS) patients. Such alterations could be conferred to mice with an intraperitoneal injection of sera from these patients or with purified immunoglobulin G. Later, we confirmed the presence of similar alterations in the superoxide dismutase 1 G93A transgenic mouse strain model of familial ALS. These consistent observations suggested that calcium plays a central role in the pathomechanism of ALS. This may be further reinforced by completing a similar analytical study of the MATs of ALS patients with identified mutations. However, due to the low yield of muscle biopsy samples containing MATs, and the low incidence of ALS patients with the identified mutations, these examinations are not technically feasible. Alternatively, a passive transfer of sera from ALS patients with known mutations was used, and the MATs of the inoculated mice were tested for alterations in their calcium homeostasis and synaptic activity. Patients with 11 different ALS-related mutations participated in the study. Intraperitoneal injection of sera from these patients on two consecutive days resulted in elevated intracellular calcium levels and increased vesicle densities in the MATs of mice, which is comparable to the effect of the passive transfer from sporadic patients. Our results support the idea that the pathomechanism underlying the identical manifestation of the disease with or without identified mutations is based on a common final pathway, in which increasing calcium levels play a central role.

## 1. Introduction

Amyotrophic lateral sclerosis (ALS) is one of the most common motor neuron diseases, which, according to the historical principles of neurology, primarily affects the upper and lower motor neurons [1]. ALS patients are traditionally sorted into two categories. Familial ALS patients represent 5–10% of the patient population, with more than a dozen, mostly autosomal dominant, mutations in different genes [2,3]. The sporadic form, with no familial accumulation, represents the overwhelming majority, i.e., 90–95%, of ALS patients [4]. However, the identification of an expansion of the intronic hexanucleotide repeat sequence in chromosome nine open reading frame 72 (C9ORF72) in patients with no family history significantly contributed to the decline of the traditional distinction between sporadic and familial ALS, which have identical clinical manifestations [5]. Since the hexanucleotide repeat sequence in C9ORF72 is now considered the most frequent genetic alteration, not just in ALS but in frontotemporal dementia [6], ALS is now considered a multisystem disorder, overlapping mostly with frontotemporal dementia [7].

In addition, regarding disease manifestations on a continuum of frontotemporal dementia, ALS blurs the distinction between two specific neurodegenerative diseases. Another noteworthy observation is that the identified pathomechanisms of ALS are not restricted to this syndrome [8]. Indeed, mechanisms including, but not restricted to, excitotoxicity [9,10], oxidative stress [11,12,13], mitochondrial dysfunction [14,15], immune/inflammatory reactions [16,17,18] and perturbed neuronal calcium homeostasis [19,20,21,22,23] are shown to contribute to the pathobiology of multiple sclerosis, Alzheimer’s, Parkinson’s, Huntington’s, and other diseases as well.

ALS plays a distinguished role since, unlike other neurodegenerative diseases affecting the central nervous system, parts of the degenerating nerve cells can be sampled without the necessity of ethically questionable brain biopsies. This special anatomical feature [24] provides the opportunity to compare and validate the results obtained from animal models with appropriate human samples.

It is now accepted that the disturbed calcium homeostasis of affected neurons plays a general role in neurodegenerative diseases [19], and particularly in ALS, this mechanism may serve as a central factor, providing positive feedback between the individual components of the complex pathomechanism of the disease [25]. In our pioneering study with electron microscopic analyses, we described an elevated calcium level accompanied by an increased number of synaptic vesicles in the motor axon terminals obtained from sporadic ALS patients [26]. While these results documented for the first time the perturbed calcium homeostasis in degenerating human nerve cells, the applied sampling was not uniform, since it was restricted to a subpopulation of ALS patients with sporadic classifications and no identified mutations. To complete the study and generalize the results, an analysis of the muscle biopsy samples from patients with identified mutations was required.

Based on our human muscle biopsy study, motor axon terminals could be localized in only 10% of the 71 patients who underwent a routine biopsy of the biceps muscles in a period of 9 months [26]. In view of these numbers, and because of the low incidence of patients with familial history or identified mutations, similar examinations are not feasible, since according to the current epidemiological data [27], the worldwide prevalence of ALS is 4–8 persons/100,000 inhabitants. Thus, such a study would require a group size of 10 million to provide the same number of patients with a familial history or identified mutations as in the sporadic study.

**Figure 1:**
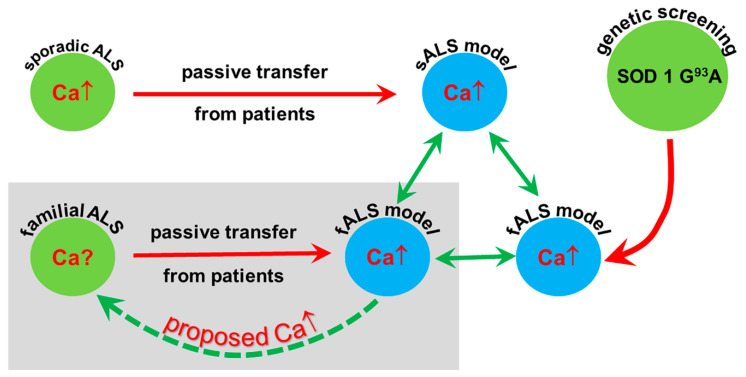
Amyotrophic lateral sclerosis (ALS) patients are represented with green circles, and the ALS models are symbolized with blue circles. With the passive transfer of serum or immunoglobulin G (IgG) from sporadic patients to mice (red arrow), a model of sporadic ALS could be created which reproduces the elevated calcium in the motor axon terminals demonstrated in the patients. ALS patients are represented with green circles, and the ALS models are symbolized with blue circles. A transgenic model of familial ALS, based on the mutations identified in patients, could also be created (curved red arrow). By replicating the sporadic ALS model with the passive transfer of sera from familial patients (shaded area), another model of familial ALS was set up in the present study. Since in each model comparable increases of calcium in the motor axon terminals could be demonstrated (green arrows), an elevated calcium level in the motor axon terminals, similar to that seen in sporadic patients, could be hypothesized for the familial ALS patients (dashed green arrow).

An indirect approach to disclose information about the underlying mechanisms in patients with ALS-related mutations relies on the passive transfer of their blood-derived samples to experimental animals. Indeed, anomalies, i.e., elevated calcium levels and increased numbers of synaptic vesicles, observed in the motor axon terminals of sporadic ALS patients, can be transferred to mice inoculated with whole serum or even with immunoglobulin G (IgG) purified from the blood of these patients [28]. If similar alterations are induced in the blood samples from patients with known mutations (mostly familial patients), assuming corresponding changes in the donor patients, a coherent view of the role of calcium in the pathomechanism of ALS could be confirmed. With the present demonstration that increased calcium can be transferred with the sera from ALS patients with identified mutations, data describing increased calcium in the axon terminals of sporadic ALS patients, increased calcium after passive transfer from sporadic patients and increased calcium in the genetic model of familial ALS could be obtained (Figure 1).

Twelve ALS patients with identified mutations, with or without family history (two patients had no family history or known mutations) were introduced to the study. Sera prepared from the patients were intraperitoneally injected into mice for 2 days with daily injections, then the motor axon terminals were analyzed electron microscopically to determine the calcium content and the number of synaptic vesicles. The observed increase in calcium levels and the elevated number of synaptic vesicles in the axon terminals, together with the previous observations of elevated calcium in the genetic model of familial ALS and the passive transfer model of sporadic ALS, provide additional data for the concept that elevated calcium levels play a central role in the pathomechanism of ALS. This common denominator may contribute to the identical clinical manifestation of the disease in its final stage.

## 2. Results

### 2.1. Ultrastructural Alterations of the Motor Axon Terminals after Inoculation with ALS Sera

**Figure 2:**
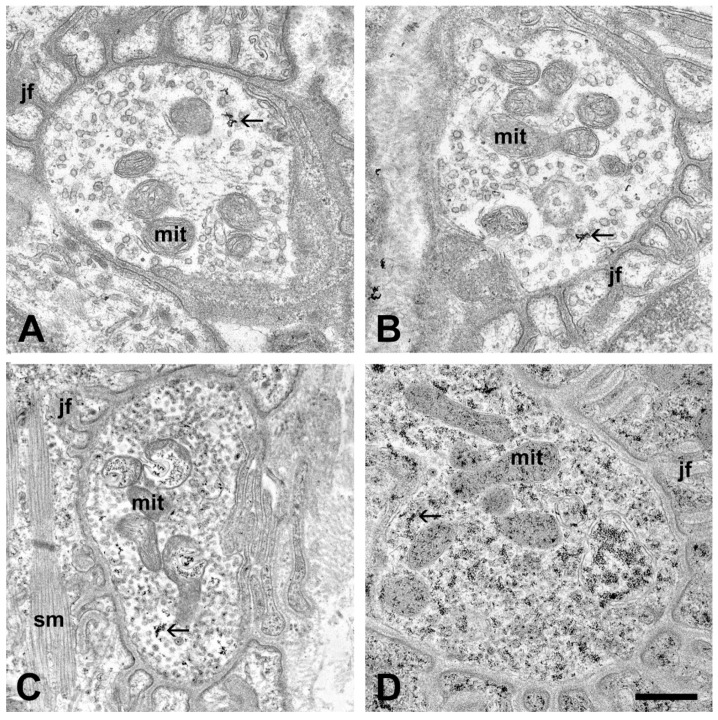
Electron micrographs of the neuromuscular synapses in the interosseous muscles after oxalate-pyroantimonate fixation. Junctional folds (jf) with no structural alterations are visible around all axon terminals. Axon terminals from an untreated mouse (**A**), and from a mouse injected with serum from a healthy individual (**B**) show no sign of structural damage, and contain intact mitochondria (mit). Furthermore, calcium-containing electron-dense deposits (EDDs) are only sparsely visible (arrows). Axon terminals from mice injected with sera from ALS patients, exemplified with superoxide dismutase 1 (SOD1) pAsp90Ala (**C**) and chromosome 9 open reading frame 72 (C9ORF72 (**D**)) mutations, display an increased amount of EDDs (arrows), particularly in (**D**). Furthermore, a global increase in synaptic vesicles can be noted. Occasionally, swollen mitochondria (mit) with clusters of EDDs are visible (**C**). sm: skeletal muscle. Scale bar: 500 nm.

**Figure 3:**
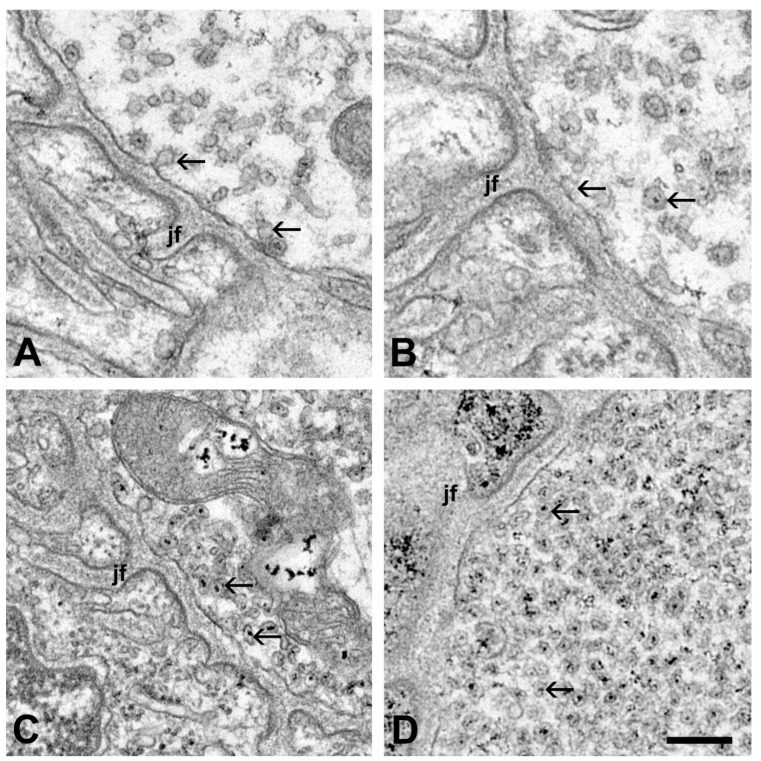
Enlarged view of motor axon terminals in the vicinity of the active zones of the neuromuscular synapses—oxalate-pyroantimonate fixation. In the axon terminal of an untreated mouse (**A**), and a mouse injected with serum from a healthy individual (**B**), only a few synaptic vesicles (arrows) are present. Some of them contain dot-like electron-dense deposits (EDDs). Axon terminals from mice injected with serum from ALS patients, represented with superoxide dismutase 1 (SOD1) pAsp90Ala (**C**) and chromosome 9 open reading frame 72 (C9ORF72) mutations (**D**), display an increased number of synaptic vesicles in these regions. The increase in the number of synaptic vesicles is exceptionally high in the axon terminal of a mouse injected with the sera from a patient with a C9ORF72 mutation (**D**). jf: junctional folds. Scale bar: 200 nm.

The postsynaptic compartments of the neuromuscular synapses of mice inoculated with sera obtained from different ALS patients displayed no structural alterations: intact muscle fibers and specialized postsynaptic membranes were visible (Figure 2). Compared to the controls, the most obvious alterations of the motor axon terminals of mice injected with sera from ALS patients were the increased number of electron-dense deposits (EDDs), representing the distribution of calcium in the tissue, and the increased number of synaptic vesicles (Figure 2 and Figure 3). Qualitatively, these changes were most prominent in the axon terminals of mice injected with sera from the patient with a C9ORF72 mutation (Figure 2D). Occasionally, swollen mitochondria containing clusters of EDDs were encountered in the axon terminals of the mice (Figure 2C).

### 2.2. Quantitative Analysis of the Change in the Number of Synaptic Vesicles and Increase in Intracellular Calcium in Motor Axon Terminals after an Inoculation with ALS Sera

**Figure 4:**
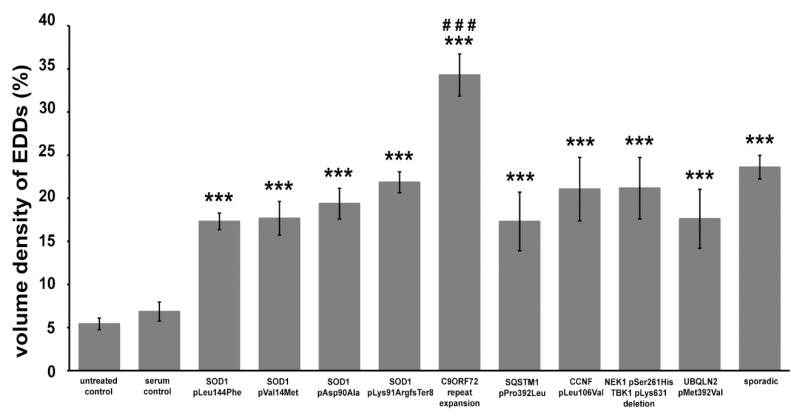
The ratio of the volume of electron-dense deposits (EDDs) and the volume of the axon terminals after inoculation with sera from ALS patients. A significant elevation in EDDs could be noted in each amyotrophic lateral sclerosis (ALS) serum treated group. Furthermore, this elevation was significantly higher (###: *p* < 0.001) in the motor axon terminals of mice injected with sera from ALS patients with C9ORF72 mutations compared to all other groups. Data are represented as the mean value ± standard error of the mean (s.e.m.). Statistical evaluation was determined using a one-way analysis of the variance (ANOVA) with the least significant difference post-hoc pairwise comparison. ***: *p* < 0.001.

The intracellular calcium level of motor axon terminals was expressed as the volume occupied by the EDDs relative to the volume of the axon terminal. Supporting the qualitative observations, an inoculation with all ALS sera induced a significant calcium increase in the axon terminals (superoxide dismutase 1 (SOD1) pLeu144Phe: 17.31% ± 0.96%; SOD1 pVal14Met: 17.67% ± 1.96%; SOD1 pAsp90Ala: 19.37% ± 1.78%; SOD1 pLys91ArgfsTer8: 21.84% ± 1.23%; C9ORF72: 34.29% ± 2.45%; sequestosome 1 (SQSTM1) pPro392Leu: 17.30% ± 3.41%; G2/mitotic-specific cyclin F (CCNF) pLeu106Val: 21.06% ± 3.68%; NEK1 (NIMA-related kinase 1) and TBK1 (TANK-binding kinase 1): 20.47% ± 3.19%; UBQLN2 (Ubiquilin 2): 17.62% ± 3.42%; sporadic: 23.60% ± 1.37%) compared to the control groups (untreated: 5.42% ± 0.68%; healthy serum treated: 6.86% ± 1.10% (Figure 4)).

**Figure 5:**
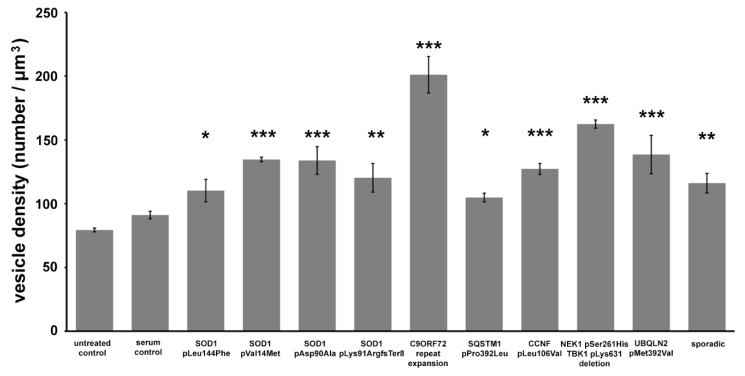
Volume density of synaptic vesicles in the active zones of neuromuscular synapses of mice inoculated with sera from amyotrophic lateral sclerosis (ALS) patients. All sera from ALS patients induced a significant increase in the number of active zone synaptic vesicles. Data are represented as the mean value ± standard error of the mean (s.e.m.). Statistical evaluation was determined using a one-way analysis of the variance (ANOVA) with the least significant difference post-hoc pairwise comparison. *: *p* < 0.05; **: *p* < 0.01; ***: *p* < 0.001.

To reduce the variability of the results due to the largely inhomogeneous distribution of synaptic vesicles within the axon terminals, a quantitative evaluation of the density of synaptic vesicles was limited to the active zones, the physiologically most relevant regions of the synapses. All treatments with ALS sera resulted in significant increases in the synaptic vesicle density (SOD1 pLeu144Phe: 110.30 ± 8.81 vesicles/µm^3^; SOD1 pVal14Met: 134.65 ± 1.82 vesicles/µm^3^; SOD1 pAsp90Ala: 133.87 ± 10.88 vesicles/µm^3^; SOD1 pLys91ArgfsTer8: 120.28 ± 11.26 vesicles/µm^3^; C9ORF72: 201.15 ± 14.29 vesicles/µm^3^; SQSTM1 pPro392Leu: 108.01 ± 12.74 vesicles/µm^3^; CCNF pLeu106Val: 124.60 ± 9.91 vesicles/µm^3^; NEK1 and TBK1: 162.43 ± 3.19 vesicles/µm^3^; UBQLN2: 138.55 ± 15.02 vesicles/µm^3^; sporadic: 116.10 ± 7.68 vesicles/µm^3^), while there were no significant changes in the group treated with healthy sera (89.40 ± 6.05 vesicles/µm^3^) compared to the control (80.38 ± 1.5 vesicles/µm^3^ (Figure 5)).

**Figure 6:**
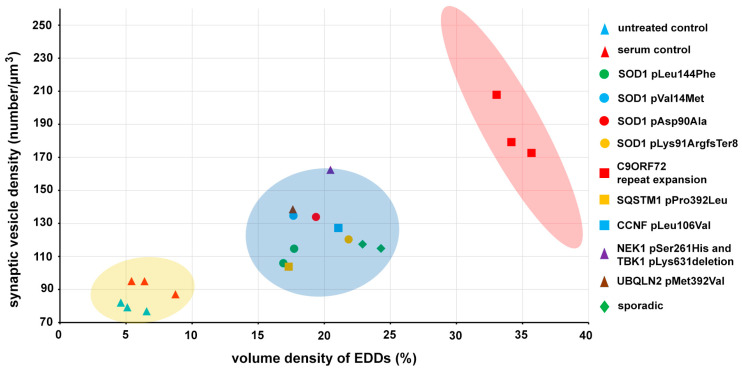
The active zone synaptic vesicle density is plotted against the volume density of the calcium-containing electron-dense deposits (EDDs). Since all amyotrophic lateral sclerosis (ALS) sera treatments resulted in a mutual increase in calcium levels and the density of synaptic vesicles, their combined values could form new groups (blue ellipse) separated from controls (yellow ellipse). The points representing the patients with the chromosome 9 open reading frame 72 (C9ORF72) mutation were sorted into a stand-alone cluster (represented by the red ellipse), and this group is beyond the 99.5% confidence regions of the control and the ALS groups represented by the covered area of the yellow and blue confidence ellipses.

The elevations in the density of the active zone synaptic vesicles and the level of intracellular calcium were noted as significant effects of the inoculation with sera from ALS patients. Since these parameters are closely associated with the activity of the synapses, their cross-relation was analyzed as well (Figure 6). In Figure 6, each point represents patients, except the “untreated control” points, which display values derived from individual mice, to illustrate the reproducibility of the histochemical method. The data points are split into three main groups: controls, ALS patients and ALS outliers, representing the patients with the C9ORF72 mutation. A K-means statistical cluster analysis confirmed the presence of these distinct clusters: (i) one represented the untreated mice and the healthy volunteers, (ii) another shows all ALS mutations, except the C9ORF72, (iii) and the last cluster represented the patients with a C9ORF72 mutation (Figure 6).

## 3. Discussion

Calcium, a ubiquitous second messenger, plays an integral physiological role as summarized by Otto Loewi in his famous saying “Yes, calcium is everything” (1959). It has also been proposed to play a role in pathological situations [29]. However, starting from the early 1980s, based on the pioneering studies of Dennis W. Choi on neocortical cultures [30,31], an autonomous “calcium hypothesis” has been developed, since, according to his observations, the “toxicity of glutamate on cortical neurons may depend primarily on the presence of extracellular calcium, probably through a mechanism which is distinct from simple excitotoxicity” [32]. Nowadays it is widely accepted that increased calcium levels are a key factor not only in acute injuries [10,33] but also in the pathobiology of neurodegenerative diseases [19,21,34,35,36,37].

The role of calcium in the pathomechanism of different neurodegenerative diseases has been directly or indirectly evidenced in their models, such as in Alzheimer’s disease [38], Parkinson’s disease [36], or ALS [39,40]. However, a direct demonstration of altered calcium levels in degenerating human nerve cells has been hampered due to the absence of brain biopsy samples for calcium studies, because of evident ethical concerns, and as autopsy samples are not suitable for this purpose since the fast diffusion of intracellular elements significantly alters their original distribution. ALS, however, is unique compared to other neurodegenerative diseases, since, due to the anatomy of the motor system, parts of the degenerating motor neurons residing in the skeletal muscles can be sampled with routine muscle biopsies [24].

In our original study, based on the results of muscle biopsies obtained from seven sporadic ALS patients, increased calcium levels and an increased synaptic vesicle number could be demonstrated in the motor axon terminals. These parameters separated the patients from the control population [26]. Similar alterations could be transferred to mice via inoculation of the total sera or IgG obtained from these patients [17,28]. In the present experiment, to prove that similar alterations may exist in the rest of the population of ALS patients, sera from ALS patients with identified mutations were collected, inoculated into mice, and the motor axon terminals in the hindlimb interosseous muscles were analyzed. For these experiments, instead of IgG, serum was used for injections, as in our previous study [17], because it exhibits more complex and stronger biological effects than the isolated and thus partially inactivated IgG. The degenerative effect of ALS serum partially comes from the presence of autoimmune IgG antibodies, which were characterized in another study where a panel of 20 IgG antibodies specific for ALS were identified from the sera of ALS patients [41]. Furthermore, purified IgG can initiate motoneuronal calcium accumulation, as was demonstrated by Pullen and his coworkers [42,43]. ALS IgG also induced selective motor neuron apoptosis in rat mixed primary spinal cord cultures [44]. Immunoglobulins from patients with sporadic ALS can bind [45] and alter the function of L-type and P-type, as well as other neuronal calcium channels [46,47,48,49]. These antibodies induce ultrastructural degeneration in motor neurons and an increase in intracellular calcium [50], as well as an increase in the frequency of miniature endplate potentials [48] and glutamate levels in the cerebrospinal fluid [51]. The intraperitoneal administration of IgG from ALS patients and the immunized goat model of ALS increased TNF-α, IL-6, and IL-10 levels in the spinal cord and the serum of inoculated mice [52]. The pathomechanistic role of ALS IgG in the sera of the patients seems to be essential since, if the IgG is removed by pre-incubating it with anti-human IgG or with the putative target of the antibodies, the pathobiological effects completely vanish [48]. Besides the effect on motor neurons, the various specific effects of ALS IgG were described in other cell types, also playing a role in the pathomechanism of ALS. These antibodies are capable of inducing oxidative stress and an upregulation of the antioxidative system in a BV-2 microglial cell line [53] and also affect cytosolic calcium homeostasis in cultured rat astrocytes [54]. Nevertheless, sera from two sporadic ALS patients were also included in the present study, which proved that identical results could be obtained from these patients as in the previous study. The present results, together with our earlier findings [26], provide the basis for the assumption that in ALS, regardless of the primary cause of the disease, calcium plays a central role in the pathomechanism. The increased number of synaptic vesicles and the simultaneously elevated levels of intra-terminal calcium are congruent with the needle electromyography findings in ALS patients with spontaneous and involuntary discharges, which may arise from the motoneuron or its axon [55]. In vitro, our findings are also compatible with the physiological abnormality of increased miniature endplate potentials, which are depolarizations of the postsynaptic terminal caused by the release of a single vesicle into the synaptic cleft, which could be induced by the passive transfer of IgG [56] or sera [57] from ALS patients.

The present findings demonstrate that, according to the mutual effect of the sera from ALS patients with known mutations on the number of synaptic vesicles and the level of intracellular calcium, the patients can be sorted into clusters that differentiate them from the controls (Figure 6). Although it lies beyond the scope of the present study to analyze the effect of the sera obtained from patients with different mutations, it is noteworthy that the effect of the sera obtained from patients with the C9ORF72 mutation exceeds the effect of all other sera from patients with identified mutations (Figure 6). The extraordinary effect of this mutation might be attributed to both the loss of function of the C9ORF72 protein and toxic gain of function from the C9ORF72 repeat ribonucleic acid (RNA), or from dipeptide repeat proteins produced by a repeat-associated non-ATG translation [58]. Mutations in this gene have a strong immune/inflammatory effect due to a generalized systemic pro-inflammatory state, which initiates autoimmunity-related degenerative pathways and microglia activation in the C9ORF72 knock-out model of ALS [59], without a regression of the motor function [60].

In summary, it should be noted that starting from the original observation that calcium is indeed elevated in the degenerating nerve cells of humans [26], which can be replicated in different models of ALS [28,61], several lines of study have been initiated, investigating this pathobiological process as a possible neuroprotective strategy in ALS. For example, based on the recognition that certain calcium-binding proteins may lend resistance to neurons [62,63,64], double transgenic parvalbumin × mutant SOD1 mice were bred, overexpressing the parvalbumin protein in spinal motor neurons, which delayed disease onset in the mice, but could not ultimately rescue them [65]. It was a reasonable assumption that increasing the calcium buffering capacity of the cells would increase their tolerance, but, per definition, sooner or later saturation occurs if the stress endures. However, an alternative therapeutic approach was based on reducing the calcium influx through calcium-permeable α-amino-3-hydroxy-5-methyl-4-isoxazole propionic acid (AMPA) receptors, which has a specific alteration in ALS [66]. After some promising results in SOD1 transgenic mice with the drug Talampanel [67], which was also introduced in clinical trials, it turned out, that its protective effect against calcium overload was effective only if the treatment was started before the appearance of clinical symptoms [68]. Our present results confirm the central role of calcium in the pathomechanism of ALS and probably in other neurodegenerative diseases. This phenomenon may unify the different pathomechanisms into a self-perpetuating cascade [25] responsible for the identical clinical manifestation of different forms of ALS, regardless of whether the patients possess mutations for the disease, which underlines the necessity of early diagnostic markers for effective treatments.

## 4. Materials and Methods

### 4.1. Ethics Approval and Consent to Participate

Ethical approvals for the studies involving animals were given by (1) The Government Office in Csongrád-Csanád County, Hungary; (2) The Committee for Animal Experiments of the University of Szeged, Szeged, Hungary I. 74-II/2015 (14 January 2015). All experiments were carried out in accordance with the institutional guidelines for the use and care of experimental animals and the governmental law for animal protection (XXVIII. chapter IV. paragraph 31) which conforms to international laws and policies (EEC Council Directive 86/609, OJL 358 1 DEC. 12, 1987; NIH Guide for the Care and Use of Laboratory Animals, United States National Research Council, revised 1996). All efforts were made to minimize animal suffering throughout the experiments.

Ethical approval for obtaining blood from patients and controls, for research purposes, and storing it anonymously with the written informed consent of patients and controls was given by The Human Investigation Review Board, University of Szeged, Hungary, in agreement with the declaration of the Medical World Federation proclaimed in Helsinki 1964. (Project title: Search for Biomarkers in Neurodegenerative Diseases: Amyotrophic Lateral Sclerosis, Parkinson’s Disease and Alzheimer’s Disease #2557/2009 (29 June 2009, revised 23 January 2012).

### 4.2. Patients

Fourteen ALS patients (Table 1) and three healthy controls participated with informed consent in the study. Five ALS patients possessed mutations in the SOD1 gene. Three patients had a GGGGCC hexanucleotide repeat expansion in the C9ORF72 gene. One patient had a mutation in the SQSTM1 gene, another one had a mutation in the CCNF gene, a further one had a mutation in the UBQLN2 gene and finally, a patient with ALS and frontotemporal dementia had a double mutation in the NEK1 gene together with a mutation in the TBK1 gene (Table 1). Two patients, composing the sporadic ALS group, were genetically screened for all 35 major ALS genes and proved to be negative (Table 1). For controls, sera from 3 age-matched healthy volunteers were obtained. Untreated animals also served as controls.

The genomic DNAs were isolated from blood samples. The entire coding region of the SOD1 genes and the flanking introns were amplified and the polymerase chain reaction products underwent a direct sequencing on an ABI 3100 sequencer (Thermo Scientific; Waltham, MA, USA) and were compared with the wild-type sequences on the Ensemble Genome Browser [69]. The patients who carried the GGGGCC repeat expansion were also screened for the “risk” haplotype, the rs3849942 variant, which was used as a marker for the “risk” haplotype for the patient and control genotypes. Rs3849942 genotyping was based on allelic discrimination assays using TaqMan chemistry (Life Technologies; Budapest, Hungary) [69]. In patient mfALS 4 in Table 1, the mutation screening revealed a mutation located in the signal peptide (M24I) together with the SOD1 pLeu144Phe mutations. Patient mALS 9 with an SQSTM1 mutation, patient mALS 10 with a CCNF mutation, patient mALS 11 with a UBQLN2 mutation, and patient mALS 12 with a NEK1 mutation, together with TBK1 mutations, were evaluated. ALS patients screened for 35 major ALS related genes with known mutations were targeted in next-generation sequencing posteriorly [17].

The diagnoses fulfilled the El Escorial revisited [55] and the Awaji [70] criteria for ALS. The patients were followed up several times and their disease progressions were recorded with the revised ALS functional rating scale (ALSFRS-R) [71]. The blood samples taken from the cubital veins were frozen, partly as whole blood and partly as sera after centrifugation, and stored until use at –80 °C. Only patient mALS12 had frontotemporal dementia. The scores of the Mini-Mental State Examination (MMSE) were in the normal range, except patient mALS 12 who had progressive frontotemporal dementia (Table 1). Initial symptoms in four patients with SOD1 mutations appeared as lower extremity weaknesses. In the fifth one, the initial symptoms involved all of the extremities. The disease in patient mALS 2 had progressed over 12 years with the mutation of SOD1 pAsp90Ala. The patient with the fast progression (6 months) had a novel heterozygous mutation (c.275_276delAA, pLys91ArgfsTer8) located in the fourth exon of the SOD1 gene and led to a frameshift with the insertion of 8 novel amino acids and the formation of a premature stop codon at the new amino acid position 99. Patient mALS 1 had a SOD1 mutation together with an angiogenin (ANG) mutation and lived one year after the onset of symptoms. Patient mfALS 3 and mfALS 4 survived for 2 years and 3 years after the appearance of the symptoms, respectively. The patients with a C9ORF72 repeat expansion had a short disease course initiated with bulbar (6, 8 and 12 months). The remaining four patients with genetic alterations also had a short (6 months) disease course. The two sporadic ALS cases without genetic alterations in the major ALS genes survived 9 months and 1 year after the diagnosis was established.

### 4.3. Passive Transfer with Human Sera and Tissue Preparation

Altogether, 48 male Balb/c mice, obtained from Charles River Appoints AnimaLab Hungary Kft. (Vác, Hungary), were injected intraperitoneally with 1 mL/day serum from ALS patients with different mutations (*n* = 3) or healthy serum (*n* = 3) for two days. All animals received sera from the same patient during the inoculation period. However, animals treated with serum from different patients with the same mutation (patients mfALS 3 and 4; patient mALS 6, 7 and 8) were pooled together, since there was no statistical difference between the effect of the sera from different patients with the same mutation. A similar pooling protocol was applied to animals treated with sera from sporadic patients (patient sALS 1,2). One group of animals did not receive an injection and was used as the untreated control (*n* = 3).

During the two-day inoculation period, animals were housed in plastic cages (5 animals/cage, at most) in a thermoneutral environment (22 ± 3 °C) and 12 h light/dark cycle with access to drinking water and regular rodent chow ad libitum. Twenty-four hours after the second serum injection, muscle samples from animals were prepared from the animals for the analytical study of calcium by an electron microscope, as described originally by Borgers and coworkers [72,73]. The method, as adapted and regularly tested for the specificity of calcium in our laboratory, ensures a good preservation of the tissue suitable for electron microscopy and results in the EDDs due to the precipitation of tissue calcium by the fixative [74,75,76,77,78].

First, animals under terminal anesthesia (avertin; 2,2,2-tribromoethanol, Merck, Darmstadt, Germany; 240 mg/kg body weight in a 1.0 mL volume i.p.) were transcardially perfused with 90 mM potassium oxalate (Merck; pH adjusted to 7.4 with KOH) followed by 3% glutaraldehyde (Polysciences Inc., Warrington, PA, USA; pH adjusted to 7.4 with KOH) containing 90 mM of potassium oxalate (pH 7.4). The hindlimb interosseus muscles were removed and fixed in the same fixative overnight (4 °C). Specimens were then rinsed in 7.5% sucrose (Molar Chemicals Kft., Budapest, Hungary) containing 90 mM of potassium oxalate (pH 7.4), postfixed with 2% potassium pyroantimonate (Merck) +1% osmic acid (Sigma; pH adjusted to 7.4 with acetic acid (Molar Chemicals Kft.)) for 2 h (4 °C). Next, specimens were rinsed in distilled water (pH adjusted to 10 with KOH) for 10 min, dehydrated in a graded series of ethanol (Molar Chemicals Kft.), processed through propylene oxide (Merck), and embedded in Durcupan ACM (Merck). Blocks were polymerized for 48 h at 56 °C. Semithin (0.3 µm) sections were cut from the blocks on an Ultracut UCT ultramicrotome (Leica, Wetzlar, Germany), etched [79] and stained [80], and then evaluated under an Eclipse 80i light microscope (Nikon, Tokyo, Japan) to identify the zones of innervation in the muscles. After trimming the blocks to the appropriate regions, to avoid personal bias in determining the fields for analysis [81], systematic random sets of ultrathin sections (50 nm) were prepared. The distance between the sections was set to 15 µm to circumvent repeated sampling of identical neuromuscular synapses during the collection of electron microscopic images. Sections were mounted on single-hole formvar coated copper grids (Electron Microscopy Sciences, Hatfield, PA, USA), contrasted with uranyl acetate (Electron Microscopy Sciences; 2% in 50% ethanol) [82] and lead citrate (Electron Microscopy Sciences; 2% in distilled water) [83].

### 4.4. Quantification of the Intracellular Calcium Levels in the Motor Axon Terminals

Ultrathin sections were examined using a JEM-1400Flash transmission electron microscope (JEOL, Tokyo, Japan). Sections from each animal were systematically screened at low magnification (500–2000×) for the presence of neuromuscular synapses until 15 axon terminals could be collected for microscopic analysis. Axon terminals were recorded as 16-bit grayscale images at an instrumental magnification of 12,000× with a 2k × 2k high-sensitivity scientific complementary metal-oxide-semiconductor camera (Matataki Flash sCMOS, JEOL) and saved in a tagged image file format. The relative volume of the axon terminals occupied by the EDDs representing the calcium precipitates was determined by point counting methods [84,85], which were modified for these unique structures and photographic conditions [75]. The recorded pictures were analyzed with the built-in modules of Image-Pro Plus (Media Cybernetics; Rockville, MD, USA) image analysis software. The tessellation of sampling points was superimposed onto each electron microscopic image, then sampling points hitting the axon terminals in each image served as reference areas and were counted. Sampling points hitting the EDDs within the reference area were counted as well. The corresponding counts obtained in the individual fields were summed up throughout the series of the identified axon terminals in each animal [86]. The appropriate ratios expressing the relative amount of the EDDs within these structures were calculated for each animal.

### 4.5. Quantification of the Density of Synaptic Vesicles

In addition to the calcium content in the axon terminals, changes in the synaptic vesicle density in the active zones were also analyzed and were expressed quantitatively. Electron microscopic images of 15 axon terminals were evaluated for each animal, where all active zones of the neuromuscular junction were quantified individually. Although the determination of the active zones for these measurements was arbitrary—by the symmetrical adjustment of a 100 nm wide and 200 nm long rectangle for the postsynaptic junction—this method was consistently applied during quantification, and adapted from the analysis of the human motor axon terminals [26]. The volume of the examined region was calculated using the area of the selected rectangle and the known section thickness. Subsequently, the number of synaptic vesicles was determined in the created cuboid. Synaptic vesicles were measured if their center was within the examined volume. Results were averaged per animal, then group averages were calculated using the individual values of the animals, which were expressed as volume densities.

### 4.6. Statistical Analysis

To determine the average volume occupied by EDDs within the axon terminals, and the synaptic vesicle density, the data derived from individual electron microscopic fields were pooled according to animals and passive transfer groups. Fifteen fields of view for each were analyzed in the motor axon terminals from each animal. Differences among the multiple means of the volume density of the EDDs were assessed by a one-way analysis of variance (ANOVA), followed by the least significant difference post-hoc test. All of the statistical analyses were performed with R (version 3.6.2, R Foundation for Statistical Computing, Vienna, Austria) and with R Studio Integrated Development Environment (version 1.1.453, RStudio Inc., Boston, MA, USA) for Windows. All data are presented as mean values ± the standard error of the means (s.e.m.). A cluster analysis was performed using the K-means clustering algorithm with WEKA data mining software (v3.8.3., Waikato, New Zealand) to investigate the cross-relationship of the volume density of EDDs and synaptic vesicles in patients with different ALS genotypes and controls. Since the analysis resulted in distinct clusters, the Jaccard similarity index was not calculated, but the 99.5% confidence regions of the control and ALS groups were evaluated and represented in Figure 6 as confidence ellipses.

## Figures and Tables

**Table 1:** Summary of the clinical data of the patients.

Patients	Age at Onset (Years)	Duration of the Disease at the Study	Initial Symptoms	Clinical Signs	ALS FRS-R	MMSE	Genetic Alteration	Family History	Therapy	Other Disease
mALS 1	63	1 year	proximal bilateral lower limb weakness	LMN, UMN	32/48	28/30	SOD1 pVal14 Met ANG Met24Ile	negative	Riluzole, Perindopril, Aspirin, Piracetam, Vinpocetine, Nebivolol	atherosclerosis, hypertension
mALS 2	75	12 years	bilateral lower limb weakness	LMN, UMN, B	19/48	29/30	SOD1 pAsp90Ala	negative	Riluzole	cervical and lumbar spondylarthrosis, hyperlipidemia
mfALS 3	29	2 years	gait disturbance	LMN, UMN	36/48	30/30	SOD1 pLeu144Phe	grandmother (fraternal)	Riluzole	-
mfALS 4	49	3 years	distal weakness of lower limbs	LMN, UMN, B, PB	25/48	28/30	SOD1 pLeu144Phe	grandmother (maternal)	Riluzole, Citalopram	depression, lumbar discs’ herniation
mALS 5	67	6 months	four limbs weakness	B, PB, UMN, LMN	39/48	30/30	SOD1 pLys91Arg fs Ter8	negative	Riluzole, Atorvastatin, Valsartan	breast cancer (irradiated 8 years ago), hypercholesterolemia, cervical and lumbar discs’ protrusion
mALS 6	68	6 months	bilateral peroneal palsy, dysarthria	LMN, B, UMN	44/48	30/30	C9ORF72 repeat expansion	negative	Alprazolam, Perindopril, Duloxetine	hyperparathyroidism (cured), generalized lipomatosis, osteoporosis, hypertension, depression
mALS 7	55	1 year	dysarthria, dysphagia	B, PB, LMN, UMN	37/48	27/30	C9ORF72 repeat expansion	negative	Riluzole, L-thyroxin	Hashimoto’s thyroiditis
mfALS 8	56	8 months	dysarthria, dysphagia	B, PB, LMN, UMN	36/48	30/30	C9ORF72 repeat expansion	mother with suspected ALS (not documented)	Riluzole, L-thyroxin	hypothyroidism
mALS 9	54	6 months	dyspnea	B, PB, LMN, UMN	40/48	30/30	SQSTM1 pPro392Leu	negative	Valsartan-HCT	hypertension
mALS 10	61	6 months	UMN, LMN lesions in the lower limbs	LMN, UMN, B	42/48	30/30	CCNF pLeu106Val	negative	Valsartan, Riluzole	hypertension, cervical and lumbar discs’ protrusion
mALS 11	65	6 months	four limbs weakness	LMN, UMN	43/48	29/30	UBQLN2 pMet392Val	negative	Riluzole	hypertension depression
mALS 12	37	6 months	four limbs weakness, dysarthria, cognitive deficit	UMN, LMN, B, PB	39/48	23/30	NEK1 pSer261His TBK1 pLys631 deletion	negative	Riluzole, Perindopril, Paroxetine	hypertension depression, frontotemporal dementia
sALS1	71	1 year	weakness of the right arm and leg (peroneal)	UMN, LMN	41/48	28/30	-	negative	Piracetam, Diclofenac, Aspirin, Perindopril, Isosorbide-mononitrate, Bisoprolol	hypertension, hypercholesterolemia, atherosclerosis, post zoster neuralgia
sALS2	74	9 months	dysarthria, dysphagia	B, UMN, LMN	39/48	26/26	-	negative	Amlodipine, Perindopril, Metoprolol, Atorvastatin, Riluzole	hypertension, hypercholesterolemia

mALS: ALS with identified mutation; mfALS: familial ALS with identified mutation; sALS: sporadic ALS; LMN: lower motor neuron; UMN: upper motor neuron; B: bulbar; PB: pseudobulbar; ALSFRS-R: ALS functional rating scale revised; MMSE: Mini-Mental State Examination.

## Abbreviations

ALS	Amyotrophic lateral sclerosis
ALSFRS-R	Revised amyotrophic lateral sclerosis functional rating scale
AMPA	α-amino-3-hydroxy-5-methyl-4-isoxazole propionic acid
ANG	Angiogenin
ANOVA	Analysis of variance
B	Bulbar
C9ORF72	Chromosome 9 open reading frame 72
CCNF	G2/mitotic-specific cyclin F
EDDs	Electron-dense deposits
IgG	Immunoglobulin G
LMN	Lower motor neuron
mALS	Amyotrophic lateral sclerosis with an identified mutation
mfALS	Familial amyotrophic lateral sclerosis with an identified mutation
MMSE	Mini-Mental State Examination
NEK1	NIMA-related kinase 1
PB	Pseudobulbar
RNA	Ribonucleic acid
sALS	Sporadic amyotrophic lateral sclerosis
s.e.m.	Standard error of the mean
SOD1	Superoxide dismutase 1
SQSTM1	Sequestosome 1
TBK1	TANK-binding kinase 1
UBQLN2	Ubiquilin 2
UMN	Upper motor neuron
